# Corticospinal and reciprocal inhibition actions on human soleus motoneuron activity during standing and walking

**DOI:** 10.14814/phy2.12276

**Published:** 2015-02-25

**Authors:** Berthe Hanna-Boutros, Sina Sangari, Louis-Solal Giboin, Mohamed-Mounir El Mendili, Alexandra Lackmy-Vallée, Véronique Marchand-Pauvert, Maria Knikou

**Affiliations:** 1 Sorbonne Universités, UPMC Univ Paris 06, UMR 7371, UMR_S 1146, LIBParis, France; 2 CNRS, UMR 7371, LIBParis, France; 3 INSERM, UMR_S 1146, LIBParis, France; 4 Sensorimotor Performance Laboratory, Konstanz UniversityKonstanz, Germany; 5 The Graduate Center, City University of New YorkNew York, New York; 6 Sensory Motor Performance Program, Rehabilitation Institute of ChicagoChicago, Illinois; 7 Department of Physical Medicine and Rehabilitation, Northwestern University Feinberg School of MedicineChicago, Illinois

**Keywords:** Humans, Ia interneurons, motor control, soleus H-reflex, TMS

## Abstract

Reciprocal Ia inhibition constitutes a key segmental neuronal pathway for coordination of antagonist muscles. In this study, we investigated the soleus H-reflex and reciprocal inhibition exerted from flexor group Ia afferents on soleus motoneurons during standing and walking in 15 healthy subjects following transcranial magnetic stimulation (TMS). The effects of separate TMS or deep peroneal nerve (DPN) stimulation and the effects of combined (TMS + DPN) stimuli on the soleus H-reflex were assessed during standing and at mid- and late stance phases of walking. Subthreshold TMS induced short-latency facilitation on the soleus H-reflex that was present during standing and at midstance but not at late stance of walking. Reciprocal inhibition was increased during standing and at late stance but not at the midstance phase of walking. The effects of combined TMS and DPN stimuli on the soleus H-reflex significantly changed between tasks, resulting in an extra facilitation of the soleus H-reflex during standing and not during walking. Our findings indicate that corticospinal inputs and Ia inhibitory interneurons interact at the spinal level in a task-dependent manner, and that corticospinal modulation of reciprocal Ia inhibition is stronger during standing than during walking.

## Introduction

Reciprocal Ia inhibition, the postsynaptic pathway mediating inhibition to antagonist motoneurons through Ia inhibitory interneurons, is a key spinal pathway for coordination of antagonist muscles activation, and is the most thoroughly studied spinal circuit in human subjects (Pierrot-Deseilligny and Burke 2012). Several research studies have delineated the amplitude modulation of the reciprocal Ia inhibition at rest and during movement. In healthy humans, reciprocal inhibition from flexor group Ia afferents on soleus motoneurons decreases during ankle plantarflexion and may increase or remain unaltered during or at the onset of ankle dorsiflexion (Shindo et al. 1984; Crone et al. 1987; Petersen et al. 1998a; Morita et al. 2001), decreases upon imposed hip angle movements (Knikou 2005), decreases during ankle co-contraction (Nielsen and Kagamihara 1992), is present when sensory afferent feedback is absent and before the onset of antagonist muscle activity (Crone and Nielsen 1989, 1994), and is modulated in a phase-dependent manner during human walking (Capaday et al. 1990, 1995; Lavoie et al. 1997; Petersen et al. 1999; Kido et al. 2004; Mummidisetty et al. 2013).

Reciprocal Ia inhibition is considered one of the major contributing segmental reflex circuits to the soleus H-reflex phase-dependent modulation during walking (Lavoie et al. 1997; Petersen et al. 1999; Ethier et al. 2003). Recordings from Ia inhibitory interneurons and lumbar motoneurons during fictive locomotion in spinal-transected cats revealed that the hyperpolarization of soleus alpha motoneurons coincides with activity of Ia inhibitory interneurons (Pratt and Jordan 1987; Degtyarenko et al. 1996; Geertsen et al. 2011). Further, Ia inhibitory interneurons are influenced by segmental interneuronal circuits, afferents, and supraspinal inputs (Eccles et al. 1956; Hongo et al. 1969; Hultborn et al. 1971, 1976), with corticospinal descending volleys to facilitate transmission in Ia inhibitory interneurons (Lundberg and Voorhoeve 1962).

The general notion is that alpha motoneurons and Ia inhibitory interneurons are activated in parallel by supraspinal centers securing a coordinated contraction of agonists and relaxation of antagonists (Lundberg 1964, 1979). Intracortical stimulation in monkeys revealed that the same interneurons mediate disynaptic inhibition of spinal motoneurons evoked by corticospinal fibers and by antagonist group Ia afferents (Jankowska et al. 1976). In humans, transcranial electrical stimulation over the foot area of the motor cortex increased reciprocal Ia inhibition (Iles and Pisini 1992), but subthreshold transcranial magnetic stimulation (TMS) failed to provide evidence for convergence of descending inputs to Ia inhibitory interneurons (Kudina et al. 1993). This was attributed to reduction of Ia inhibition by actions of the opposite Ia inhibitory interneurons (Rothwell et al. 1984).

Collectively, the main objective of this study was to assess the effects of corticospinal inputs on soleus H-reflex excitability and reciprocal Ia inhibition during standing and walking in healthy humans. We hypothesized that reciprocal Ia inhibition is adjusted based on the motor task and phase of walking, and that corticospinal input affects reciprocal inhibition differently during standing and walking.

## Methods

### Subjects

Experiments were performed in 15 healthy subjects (11 females, age range 22–44 years, 28.5 ± 1.8 years), all of whom gave informed written consent to the experimental procedures before participation to the study, which were approved by the ethics committee of the Pitié-Salpêtrière Hospital (CPP Ile de France VI). Subjects’ consent and study procedures conformed to the standards set by the Declaration of Helsinki.

### Recordings

Activity of soleus and tibialis anterior (TA) muscles on the right side was recorded with bipolar surface electrodes (ZeroWire EMG, Aurion Srl, Milan, Italy). For the soleus muscle, the electrodes were placed medially on the posterior aspect of the leg, 2–3 cm below the gastrocnemius muscles. For the TA muscle, the electrodes were placed on the anterior aspect of the leg, 10–15 cm below the patella. EMG activity was filtered (EMG bandwidth 10–500 Hz) and amplified (×1,000) before being digitally stored (2,000 Hz sampling rate) on a personal computer for offline analysis (Power 1401 and Signal Software, CED, Cambridge, UK).

### Peripheral nerve and cortical stimulation

#### Posterior tibial nerve (PTN) stimulation

The right PTN was stimulated with 1-msec rectangular electrical pulses delivered through surface electrodes via a constant current stimulator (DS7H, Digitimer Ltd, Hertfordshire, UK). A 7-cm^2^ brass hemispheric electrode was placed at the popliteal fossa (cathode), and a 21-cm^2^ brass plaque was placed proximal to the patella (anode). The optimal stimulation site corresponded to the one that an H-reflex could be evoked without an M-wave at low stimulation intensities, and the H-reflex had a similar shape to the M-wave at increased stimulation intensities (Knikou 2008).

#### Deep peroneal nerve (DPN) stimulation

DPN stimulation was used as a conditioning stimulus for the soleus H-reflex. Stimulation was delivered via two 7-cm^2^ brass hemispheric surface electrodes, placed distal to the head of the fibula. The optimal stimulation site corresponded to the one that at increased levels of stimulation intensities, selective ankle dorsiflexion without ankle eversion was present (Knikou 2008; Knikou and Mummidisetty 2011). The intensity of DPN stimulation was set at 1.1–1.2 times the TA M-wave motor threshold during standing and walking because the amount of reciprocal inhibition depends on the conditioning stimulation strength (Petersen et al. 1998a,b).

#### Transcranial magnetic stimulation (TMS) of M1

**Figure 1:**
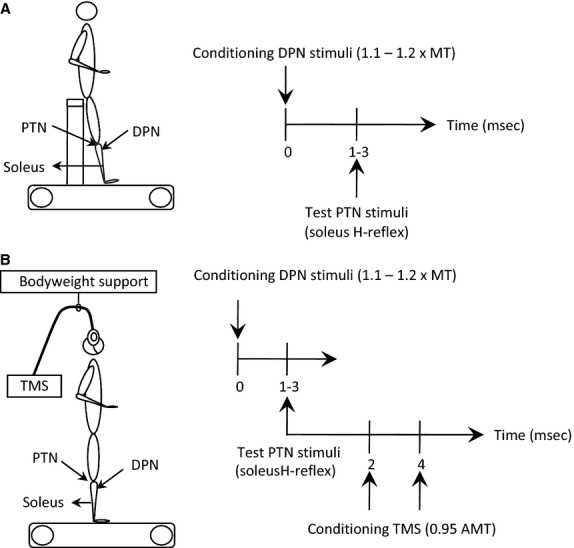
Experimental design. (A) Experimental position and conditioning-test (C-T) interval between posterior tibial (PTN, test stimuli) and deep peroneal (DPN, conditioning stimuli) nerves used to establish reciprocal inhibition of the soleus H-reflex with subjects seated. (B) Experimental position and C-T intervals between conditioning transcranial magnetic stimulation (TMS) and reciprocal inhibition on soleus H-reflex (triple stimulation paradigm).

TMS of the left M1 was also utilized as a conditioning stimulus for the soleus H-reflex. TMS was delivered through a double cone coil (Magstim Rapid, Whitland, UK) held over the longitudinal fissure at a position that induced a motor-evoked potential (MEP) in the right soleus muscle in three out of five consecutive single TMS pulses of 1-msec duration. A customized helmet made with thermoplastic plaque modeled on the coil was used to maintain the position of the coil over the head with a chinstrap. The coil cable was held by an elastic restraint, which was fixed to the treadmill unweighing system (Fig.1B, *left graph*). This reduced the coil weight and ensured a stable position of the magnetic coil. The active motor threshold (AMT) of the soleus MEP was established during standing and at mid- and late stance phases of walking. AMT corresponded to the intensity that an MEP in the soleus EMG ≥ 50 *μ*V was present, and could be evoked in more than five out of 10 consecutive single TMS pulses. TMS intensity was adjusted at 0.95 times the AMT obtained during standing and at mid- and late stance phase during walking upon TMS conditioning of the soleus H-reflex and reciprocal inhibition. At 0.95 times the AMT, descending motor volleys induce spinal cord potentials, but these volleys are at the subliminal fringe for soleus alpha motoneurons and MEPs (Kaneko et al. 1996; Lackmy-Vallee et al. 2012).

## Experimental Procedures

With subjects seated at rest, the short-latency soleus H-reflex depression induced by DPN stimulation was first determined at conditioning-test (C-T) intervals ranging from 0 to 3 msec (Fig.1A) (Crone et al. 1987). The C-T interval at which the soleus H-reflex was most depressed was utilized to assess the modulation of reciprocal inhibition during standing and walking (Mummidisetty et al. 2013), and ranged from 1 to 3 msec across subjects (see Results).

Then, the soleus maximal M-wave (Mmax) was elicited and measured as peak-to-peak amplitude during standing. The stimulation intensity to the PTN was adjusted to evoke a control H-reflex on the ascending phase of the recruitment curve that ranged from 25 to 35% *M*_max_ across subjects during standing and walking (Crone et al. 1990). Having established the optimal stimulation intensities for PTN and DPN, the C-T interval for reciprocal Ia inhibition, and the 0.95 AMT following TMS, separate or combined TMS and DPN conditioning stimuli for the soleus H-reflex were delivered to each subject during standing and randomly at the mid- and late stance phases during walking. For each subject and motor task (standing/walking), the following four types of H-reflexes were randomly recorded: (1) control soleus H-reflexes; (2) soleus H-reflexes conditioned by DPN stimulation at the optimal C-T interval of reciprocal inhibition; (3) soleus H-reflexes conditioned by TMS at the C-T intervals of −4 and −2 msec (Kudina et al. 1993; Petersen et al. 1998a) to assess corticospinal inputs on soleus H-reflex; and (4) soleus H-reflexes conditioned by DPN and TMS to assess interaction of antagonist muscle afferents and corticospinal inputs on soleus H-reflex. For each stimulation protocol, 20 H-reflexes were recorded. The effects of combined TMS and DPN stimulation were also investigated during standing at a constant C-T interval between DPN and PTN, while the C-T interval between TMS and PTN stimulation ranged from −4 to 4 msec in 1-msec incremental steps, in three out of 15 subjects. This was done in order to verify that at the C-T intervals of −4 and −2 msec between TMS and PTN tested in all subjects were optimal.

**Figure 2:**
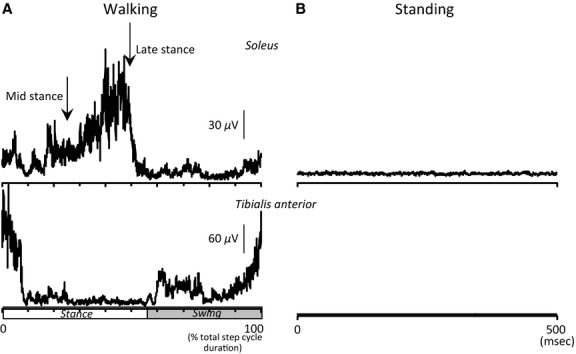
Background EMG activity. Mean rectified EMG activity in soleus (upper traces) and TA (lower traces) muscles during walking (A) and during standing (B) in one subject. Abscissa was expressed as a percentage of the total duration of the step cycle in A and in msec in B. Vertical arrows indicate when stimuli were delivered at mid- and late stance phases of walking.

During walking on a treadmill (Biodex Medical Systems Inc., Shirley, NY), a pressure transducer was placed on the right heel in order to detect the time of heel contact. Subjects walked for 5 min to accustom themselves to treadmill walking and determine the preferred treadmill speed (2.8–4.2 km/h; 3.6 ± 0.1, mean ± standard error). The different treadmill speeds resulted in varying durations of the stance phase during walking between subjects. To counteract this difference among subjects, the step cycle duration was determined from 20 consecutive steps based on the timing of the heel contact. Further, the rectified and averaged soleus EMG signals were visually inspected and the duration of the onset and offset EMG burst was determined as a function of the step cycle duration. Stimulations were delivered at mid- and late stance phase of walking at delays corresponding to activation and deactivation of soleus EMG (Fig.2). On average, stimulations were delivered 248.0 ± 15.0 msec (for midstance) and 566.0 ± 18.2 msec (for late stance) after heel contact, respectively. This corresponded to 20.9 ± 1.2% and 46.5 ± 0.7% of the total duration of the step cycle, respectively (Fig.2).

### Offline data analysis

Soleus H-reflexes and M-waves were measured as peak-to-peak amplitude. Soleus M-waves were normalized to the Mmax, while conditioned H-reflexes were normalized to control reflex values. Differences between conditioned and mean control H-reflexes, both expressed as a percentage of the mean amplitude of the control H-reflex, were used to evaluate the level of reciprocal inhibition, the effects of corticospinal inputs on the soleus H-reflex, and the effects of combined stimuli. The algebraic sum of the effects of separate stimuli was also estimated to evaluate the net effect on soleus motoneurons, and was compared with the effects evoked following combined stimuli. To establish the background EMG activity level, the mean amplitudes of the rectified band-pass filtered soleus and TA EMG at 50 msec before stimulation for a duration of 30 msec during standing and at similar delays for mid and late stance were measured. The soleus and TA background EMG activity was normalized to the maximal soleus and TA activity during walking, and the ratio between TA and soleus background activity was calculated to estimate the level of co-contraction for each motor task. Mean values are indicated ± 1 SEM.

### Statistics

The data were analyzed at three levels of interest: (1) to test the influence of conditioning stimuli on the soleus H-reflex in each individual (comparison between the 20 control H-reflexes and the 20 conditioned H-reflexes using paired *t* test); (2) to determine if the mean effect in the group was significantly different from 0 (comparing the mean effect in the 15 subjects to a theoretical value using *t* test; in this case 0 indicates no effect); and (3) to determine whether the effects of conditioning (isolated and combined) stimuli changed across tasks taking into account all the factors that could have influenced the results (multiple comparisons). Before multiple comparisons, we tested the changes in the level of soleus and TA background EMG activities, soleus and TA ratios, soleus M-waves, soleus control H-reflexes, and TMS intensities (AMT) which could have influenced the effects of conditioning stimuli across tasks. For this, because of non-normal data and variance distribution, we used Friedman tests and when a significant *P* value was found, post hoc Bonferroni Dunn's tests were performed. Correlation analysis was also performed to evaluate the relationship between the soleus background EMG activity and the H-reflex control size, and the relationship between the level of reciprocal Ia inhibition and the corticospinal facilitation.

Analysis of covariance (ANCOVA) was also performed to compare the H-reflex control size between tasks taking into account the background activity. Last, multiple regression analyses were performed to compare the effect of isolated DPN stimuli, isolated TMS, or combined stimuli on soleus H-reflex across tasks taking into account the level of soleus and TA background activity, the size of the control soleus H-reflex, and TMS intensity. Statistical analysis was conducted using StatEL software (www.adscience.eu). In all statistical tests, significant differences were tested at 95% of the confidence level.

## Results

### Reciprocal Ia inhibition during seated

**Figure 3:**
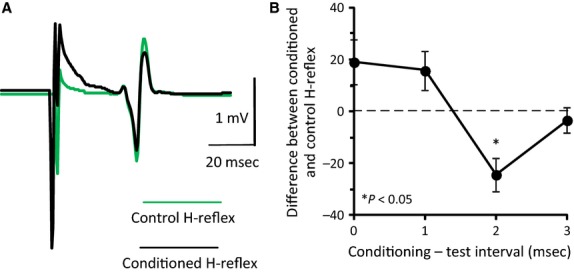
Reciprocal Ia inhibition at rest. (A) Nonrectified waveform averages (*N* = 20) of the control (green line) and conditioned (black line) H-reflexes from one representative subject during seated. Conditioning stimuli were applied to the deep peroneal nerve (DPN) at 1–1.5 × MT at a conditioning-test (C-T) interval of 2 msec. (B) Difference between conditioned and mean control H-reflex (% the mean control H-reflex) in the same subject as in A, plotted against the C-T interval between DPN and PTN stimuli. Vertical bars are ±1 SEM. *P <* *0.05.

Nonrectified waveform averages (*N* = 20) of the soleus H-reflex under control conditions and following DPN stimulation from one representative subject when seated are shown in Fig.3A. The corresponding difference between the mean amplitude of the control and conditioned soleus H-reflexes at each C-T interval tested is shown in Fig.3B. DPN stimulation significantly depressed the soleus H-reflex at 2 msec (*P *<* *0.05). On average, the C-T interval at which DPN stimulation depressed the soleus H-reflex significantly was 2.1 ± 0.1 msec: 2 msec in 11/15 subjects, 3 msec in 3/15, and 1 msec in the last remaining subject.

### Comparison of EMG background activities, compound muscle potentials in soleus, and TMS intensity during standing and walking

Because the following factors could have influenced the effects of conditioning stimuli on soleus H-reflex, we first examined their changes during tasks before multiple comparison analyses.

#### EMG background activity

Friedman tests revealed that the levels of background EMG activity in soleus and TA significantly changed between tasks (*F* = 17.7 and *F* = 15.6 for soleus and TA, respectively; *P *<* *0.001 for both muscles; Table1). Bonferroni-Dunn's post hoc analyses indicated no significant difference between the mid- and late stance phase of walking (*P *=* *0.18 and 0.15 for soleus and TA, respectively), and a significantly smaller activity during standing compared to walking for both muscles (midstance vs. standing *P *<* *0.01 and late stance vs. standing *P *<* *0.001). However, the ratio between the level of activity in soleus and TA muscles did not change during standing and walking (Friedman, *F* = 0.31, *P *=* *0.42; Table1).

**Table 1:** Parameters of neuronal excitability.

	Midstance	Late-stance	Standing
Soleus EMG*	44.8 ± 5.6	54.4 ± 6.1	20.3 ± 2.4
TA EMG*	11.9 ± 1.9	15.6 ± 2.0	5.2 ± 1.3
Ratio TA/Soleus	0.32 ± 0.06	0.31 ± 0.03	0.27 ± 0.05
M-wave	3.2 ± 0.7	3.7 ± 0.9	2.4 ± 0.8
H-reflex*	34.9 ± 2.7	37.8 ± 5.1	24.9 ± 3.1
AMT*	44.1 ± 1.2	44.7 ± 1.7	50.3 ± 1.9

Soleus and TA EMG: Mean soleus and TA background EMG activity corresponding to each tested position (expressed as % maximal EMG activity during the gait cycle recorded in each muscle). Ratio TA/soleus: ratio between the TA and soleus background EMG activities. M-wave: Mean amplitude of soleus M responses expressed as a percentage of the Mmax. H-reflex: Mean amplitude of the soleus control H-reflex expressed as a percentage of the Mmax. AMT: Mean TMS intensity (% maximal stimulator output) at AMT for each position and phase of walking.

* *P *<* *0.05 among tested conditions based on Bonferroni-Dunn's post hoc analyses for pair-wise comparisons.

#### Compound muscle potentials in soleus

While the size of the M-wave was constant across tasks (Friedman test, *F* = 0.13, *P *=* *0.94), the soleus H-reflex control size changed significantly (Friedman test, *F* = 5.25, *P *<* *0.01; Table1). A significant correlation was found between the soleus background activity and the H-reflex control size (Pearson correlation analysis, *R*^2^ = 0.62, *P *<* *0.001). By neutralizing the influence of the background EMG on the soleus H-reflex, differences in reflex size between motor tasks were not found (ANCOVA, *F* = 1.07, *P *=* *0.92).

#### TMS intensity

The AMT for evoking an MEP was significantly different between tasks (Friedman test, *F* = 14.5, *P *<* *0.001; Table1), and was higher during standing compared to walking (Bonferroni-Dunn's post hoc analyses, mid vs. late stance *P *=* *0.79, midstance vs. standing *P *<* *0.01 and late stance vs. standing *P *<* *0.05).

### Soleus H-reflex suppression by DPN stimulation during standing and walking

**Figure 4:**
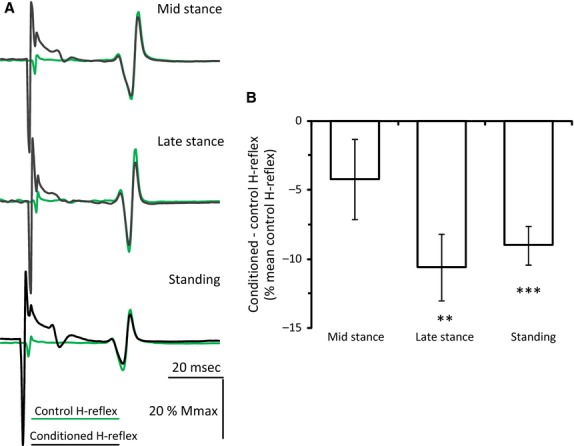
Reciprocal inhibition during standing and walking. (A) Nonrectified waveform averages (*N* = 20) of the control (green lines) and conditioned (black lines) soleus H-reflexes from one subject at midstance (upper traces) and late stance (middle traces) phases of walking, and during standing (lower traces). Conditioning stimuli were applied to the deep peroneal nerve (DPN) at 1–1.2 × MT and at a conditioning-test interval of 2 msec. (B) Overall mean difference between conditioned and mean control H-reflex (% the mean control H-reflex) for each task from all subjects. **P <* *0.01 and ***P <* *0.001. Vertical bars are ±1 SEM.

Nonrectified waveform averages of the control and conditioned soleus H-reflexes in one representative subject at mid- and late stance phases and during standing are indicated in Fig.4A. DPN stimulation reduced significantly the soleus H-reflex size at late stance and during standing in this subject (*P *<* *0.05). Across all subjects (Fig.4B), the mean soleus H-reflex depression observed at the midstance phase of walking was small and was not significant (−4.2 ± 2.9%, *P *=* *0.16). In contrast, the soleus H-reflex depression was significant at late stance (−10.6 ± 2.4%, *P *<* *0.01) and during standing (−9.0 ± 1.4%, *P *<* *0.001). To further determine whether the mean level of reciprocal inhibition changed across motor tasks, multiple linear regression analysis was performed to test the difference between tasks taking into account background EMG activities and the soleus H-reflex control size. The regression was not significant (*R*^2^ = 0, *P *=* *0.69) suggesting that reciprocal inhibition did not significantly change between tasks when taking into account the background EMG activities and the soleus H-reflex control size (soleus EMG: *P *=* *0.76; TA EMG: *P *=* *0.96; H-reflex control size: *P *=* *0.83; motor task: *P *=* *0.32).

### Soleus H-reflex modifications by TMS during standing and walking

**Figure 5:**
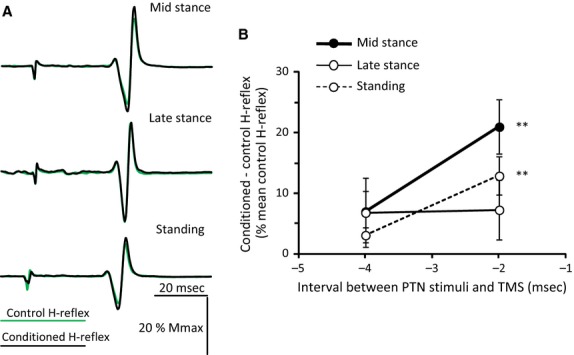
Effects of subthreshold TMS on soleus H-reflex during standing and walking. (A) Nonrectified waveform averages (*N* = 20) of the control (green lines) and conditioned (black lines) soleus H-reflexes from one subject at midstance (upper traces) and late stance (middle traces) phases of walking, and during standing (lower traces). TMS conditioning stimuli were set at 0.95 × AMT and the conditioning-test (C-T) interval was −2 msec. (B) Overall mean difference between conditioned and control H-reflex (% of the mean control H-reflex) plotted against the C-T interval between the TMS and PTN stimuli, at midstance (black line and filled circles), late stance (thin line and open circles) and during standing (interrupted line and open circles) from all subjects tested. Vertical bars are ±1 SEM. **P <* *0.01.

The waveform averages of the control and TMS-conditioned H-reflexes during standing and walking (same subject as in Fig.3A) at the C-T interval of -2 msec are indicated in Fig.5A. In this subject, TMS facilitated the soleus H-reflex at midstance and during standing (*P *<* *0.05). The overall mean difference between control and conditioned reflexes at the C-T intervals of −4 and −2 msec from all subjects is indicated in Fig.5B. In all subjects, TMS did not induce any significant changes on the soleus H-reflex at the C-T interval of −4 msec (midstance: 7.0 ± 3.4%, *P *=* *0.06; late stance: 6.8 ± 5.7%, *P *=* *0.25; standing: 3.1 ± 1.3%, *P *=* *0.05). In contrast, TMS delivered at −2 msec significantly increased the soleus H-reflex at midstance and during standing (21.0 ± 4.5 and 12.9 ± 3.2%, respectively; *P *<* *0.01). TMS-induced soleus H-reflex facilitation was small at late stance and did not reach a statistically significant level (7.3 ± 4.9%; *P *=* *0.16). Based on these findings, the level of corticospinal facilitation at the C-T interval of −2 msec between tasks was compared while taking into account the background EMG activity, the control H-reflex size, and TMS intensity. Multiple linear regression was not significant (*R*^2^ = 0.05, *P *=* *0.21) suggesting that TMS-induced soleus H-reflex facilitation did not change across tasks when taking into account the background EMG activities, the soleus H-reflex control size, and the TMS intensity (soleus EMG: *P *=* *0.67; TA EMG: *P *=* *0.53; control H-reflex size: *P *=* *0.06; TMS intensity: *P *=* *0.19; motor task: *P *=* *0.09).

### Soleus H-reflex modifications after combined conditioning stimuli

**Figure 6:**
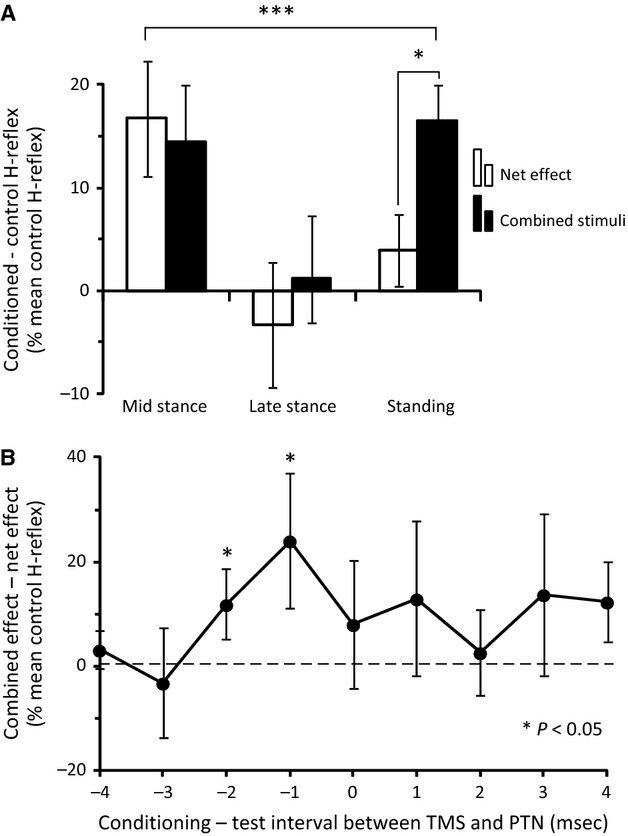
(A) Comparison between the net and combined stimuli effects. Difference between conditioned and control H-reflex (% of the mean control H-reflex) during walking (mid- and late stance phases) and standing from all 15 subjects tested. White columns illustrate the algebraic sum of the effects of separate stimuli, and black columns illustrate the effect of combined stimuli. Asterisks show statistically significant differences between the effect of combined stimuli and the net effect as well as across tasks. (B) Overall amplitude of the combined effects having subtracted the net effects is plotted against the conditioning-test interval between TMS and PTN for three subjects during standing. Vertical bars are ±1 SEM. *P <* *0.05 and ***P <* *0.01.

To further investigate the convergence of descending inputs on soleus motoneurons and Ia inhibitory interneurons during standing and walking, we compared the algebraic sum of separate DPN and TMS stimuli effects on the soleus H-reflex (net effects) to the effects evoked following combined DPN and TMS. Figure6A indicates the net and combined effects for each task from all subjects. During walking, the combined effect of TMS and DPN stimulation on the soleus H-reflex was not significantly different from the net effect evoked by separate stimuli (*P *=* *0.37 and 0.23 for mid and late stance, respectively). During standing, combined TMS and DPN stimulation facilitated the soleus H-reflex to a level that was significantly greater than the facilitation calculated by summing the effect of separate stimuli (*P *<* *0.05) (Fig.6A).

To further examine the extra facilitation across tasks, multiple regression analysis was performed to test its modifications taking into account the level of change in soleus H-reflex after TMS only (corticospinal facilitation) and DPN stimulation only (reciprocal inhibition). The effect of combined stimuli on the soleus H-reflex was significantly influenced by these parameters (*R*^2^ = 0.27, *P *<* *0.001), especially by the motor task (*P *<* *0.05) and TMS-mediated soleus H-reflex facilitation (*P *<* *0.01). However, the level of reciprocal inhibition did not influence the results (*P *=* *0.72). The results of the multiple regression analysis confirmed that the effect of combined stimuli significantly changed based on the motor task, with soleus H-reflex extra facilitation to be evident during standing. Further, the extra facilitation upon combined stimuli depended on the corticospinal control on soleus H-reflex but not on the level of reciprocal inhibition. A significant negative correlation between reciprocal inhibition and soleus H-reflex corticospinal facilitation (Pearson correlation analysis, *R*^2^ = 0.50, *P *<* *0.001) was found, suggesting that reciprocal inhibition was reduced when TMS-mediated soleus H-reflex facilitation was increased.

To verify the timing of the TMS effects (corticospinal facilitation and extra facilitation on combined stimuli), the C-T interval between PTN and TMS ranged from −4 to 4 msec in three subjects while standing. Figure6B shows the overall amplitude of the combined effects having subtracted the net effects with respect to the C-T interval. An extra facilitation of the soleus H-reflex was observed at the C-T interval of −2 and −1 msec (*P *<* *0.05).

## Discussion

The TMS-mediated soleus H-reflex facilitation, reciprocal Ia inhibition, and TMS-mediated effects on reciprocal Ia inhibition were modulated in a phase- and task-dependent manner. During standing, combined transcranial magnetic and TA group Ia afferents stimulations produced larger soleus H-reflex facilitation than that evoked by separate stimuli. During walking, the effect of combined stimuli was not different from the algebraic summation of the effects produced by separate stimuli. These findings support that Ia inhibitory interneurons are susceptible to descending inputs more during standing than during walking.

TMS over the contralateral M1 in seated subjects induces short-latency (−5 to −1 msec) soleus H-reflex facilitation that is followed 10 msec later by a period of long-lasting inhibition (Iles and Pisini 1992; Kudina et al. 1993; Nielsen and Petersen 1995; Petersen et al. 1998a). This bimodal TMS-induced soleus H-reflex excitability pattern remains unaltered during walking, but the long-lasting inhibition is replaced by facilitation during standing and tonic ankle plantarflexion (Nielsen and Petersen 1995; Petersen et al. 1998a). Further, subthreshold TMS produces suppression of the ongoing soleus and TA EMG activity during walking at a longer latency (∼40 msec; Petersen et al. 2001). Based on these findings, it was suggested that soleus and TA motoneuron activity during walking is concomitantly influenced by descending inputs.

In this study, we found that TMS-mediated soleus H-reflex facilitation was absent at late stance (Fig.B), but was present at midstance and during standing when the soleus muscle was either minimally active or completely silent (Fig.2). The smaller amplitude of the control soleus H-reflex during standing than during walking (Table1) might be partly due to different soleus background EMG activity at mid- and late stance phases, but the latter did not influence the TMS-induced soleus H-reflex facilitation. Because TMS was delivered at subthreshold levels for MEP, intracortical interneurons might have affected the corticospinal inputs to spinal motoneurons (Davey et al. 1994). Such intracortical inhibition has been observed during tonic contraction and during walking (Petersen et al. 2001), and might have been compensated by enhanced cortical excitability during walking (Petersen et al. 1998a).

The reciprocal inhibition of soleus motoneurons by the flexor group Ia afferents was significant during standing and at the late stance phase (Fig.4B), consistent with findings reported in the literature (Lavoie et al. 1997; Petersen et al. 1999; Mummidisetty et al. 2013). One may consider that this neural adaptation is the result of peripheral movement-related inputs (reafference). However, the strength of reciprocal inhibition in soleus motoneurons can be controlled independently from the level of motor activity in the ankle muscles (Capaday et al. 1990; Lavoie et al. 1997; Kasai et al. 1998; Petersen et al. 1999). This was clearly evident in our results, during which reciprocal inhibition was adjusted differently at mid- and late stance phases (Fig.4B), while the soleus background activity was similar at these phases (Table1). Thus, the smaller soleus background activity during standing compared to walking cannot account as a sole mechanism for the increased reciprocal inhibition.

Spinal segmental neuronal pathways and/or mechanisms that may have affected the reciprocal Ia inhibition are the nonreciprocal Ib inhibition, presynaptic inhibition of soleus Ia afferents, and presynaptic modulation of Ia inhibitory interneurons. This thesis is supported by the (1) presynaptic control of the flexor group Ia terminals mediating disynaptic reciprocal inhibition and monosynaptic excitation of motoneurons during locomotion (Enríquez-Denton et al. 2000; Baret et al. 2003); (2) facilitation of monosynaptic transmission by tonic modulation of presynaptic inhibition (Dueñas and Rudomin 1988; Gossard et al. 1991; Gosgnach et al. 2000; Menard et al. 2003); and (3) modulation of Ib inhibition during standing and walking in healthy humans (Stephens and Yang 1996; Marchand-Pauvert and Nielsen 2002; Faist et al. 2006). Renshaw cells activated by motor axons from ankle plantarflexors likely had no effect on Ia inhibitory interneurons, since recurrent inhibition in the TA muscle is similar during standing and walking (Lamy et al. 2008).

A key finding of the present study is that the effects of combined subthreshold TMS and TA group I afferent stimulation on soleus motoneurons were not of the same amplitude when compared to the effects produced by the same inputs delivered separately. During standing, combined stimulation produced an extra facilitation to the soleus H-reflex that was not evident upon algebraic summation of the effects following separate conditioning stimulation (Fig.6A). Further, the soleus H-reflex facilitation by combined stimuli during standing was of similar strength to that observed at the midstance phase of walking upon isolated TMS (compare Figs.5B and 6A), which coincided with small reciprocal inhibition (Fig.4B). In contrast, the TMS-mediated soleus H-reflex facilitation was larger at midstance than during standing (Fig.4B). Therefore, an increase in corticospinal facilitation alone cannot account for the extra facilitation observed during standing. Moreover, the extra facilitation was observed at a C-T interval of −2 msec between DPN and TMS (Fig.6A). At this interval, an extra facilitation of the soleus H-reflex during standing was present even when the net effects were subtracted from the effects of combined stimuli (Fig.6B). Changes in cortical excitability after DPN stimulation could not have contributed to the extra facilitation of the soleus H-reflex upon combined TMS and DPN stimulation during standing because such transcortical facilitation is observed at longer intervals (more than 50 msec; Christensen et al. 1999). During walking, the effect of combined stimuli did not differ from the algebraic summation of the effects produced by separate stimuli. The absence of an extra reflex effect upon separate stimuli suggests that interactions between corticospinal inputs and Ia inhibitory interneurons at mid- and late stance phases of walking are not strong.

**Figure 7:**
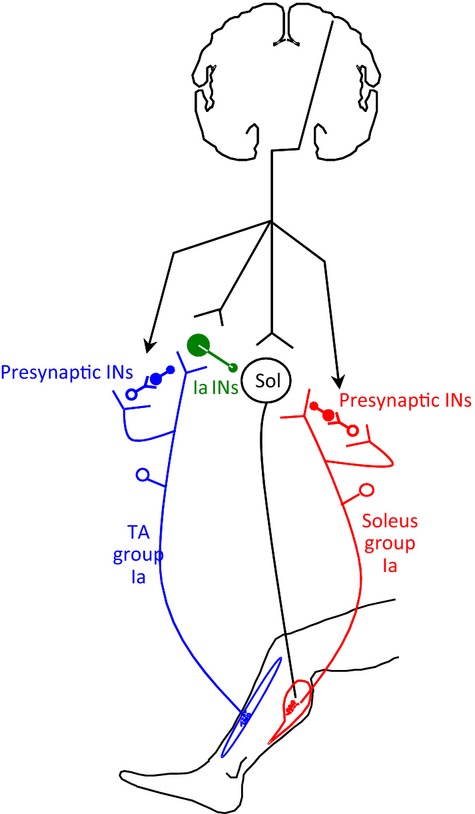
Corticospinal projections on soleus motoneurons, Ia and presynaptic interneurons. Schematic diagram showing the descending inputs from motor cortex onto soleus motoneurons (open circle with Sol inside), Ia inhibitory interneurons (in green) receiving group Ia afferents from TA (in blue), and primary afferent depolarization interneurons mediating presynaptic inhibition of Ia afferents projecting on soleus motoneurons (in red) or on Ia afferents from TA projecting on Ia inhibitory interneurons (in blue). INs = interneurons.

To summarize, the TMS-induced soleus H-reflex facilitation during standing may be due to descending activation of soleus motoneurons. At the midstance phase of walking, the TMS-induced soleus H-reflex facilitation may be due to descending activation of soleus motoneurons and concomitant descending inhibition of Ia inhibitory interneurons (because the reciprocal inhibition was reduced at midstance), and adjustments made by the spinal central pattern generator exerted as increased presynaptic control of inhibitory Ia interneurons. This presynaptic control of Ia interneurons may be potent when there is a substantial corticospinal inflow produced with TMS over M1. It should be noted that if TMS had a similar effect on the presynaptic control of monosynaptic Ia excitation and reciprocal inhibition, then this would have induced less TMS-mediated facilitation of the soleus H-reflex during standing than during walking, an effect that was apparent in this and other studies (Petersen et al. 1998a). The soleus H-reflex extra facilitation upon combined TMS and DPN stimulation may be due to descending activation of soleus motoneurons and concomitant descending inhibition of Ia inhibitory interneurons during standing (Fig.7). During walking, the central pattern generator might have occluded these effects by maximizing the phasic modulation of presynaptic inhibition and minimizing the corticospinal influences on Ia inhibitory interneurons (Fig.7).

## Conclusion

The extra facilitation of the soleus H-reflex following combined conditioning stimuli was larger during standing than during walking when compared to the algebraic sum of separate conditioning stimuli. The TMS-induced soleus H-reflex facilitation during standing likely resulted from descending activation of soleus motoneurons and concomitant descending inhibition of Ia inhibitory interneurons. During walking, the effect of combined stimuli did not differ from the algebraic summation of the effects produced by separate stimuli. These findings suggest that segmental reflex circuits are susceptible to descending inputs more during standing than during walking and that activation of soleus motoneurons is accompanied by less reciprocal inhibition.
